# Identifying common components across biological network graphs using a bipartite data model

**DOI:** 10.1186/1753-6561-8-S6-S4

**Published:** 2014-10-13

**Authors:** EJ Baker, C Culpepper, C Philips, J Bubier, M Langston, EJ Chesler

**Affiliations:** 1 Department of Computer Science, Baylor University, Waco, TX, USA; 2 Department of Electrical Engineering and Computer Science, University of Tennessee, Knoxville, TN, USA; 3 Department of Bioinformatics and Computational Biology, The Jackson Laboratory, Bar Harbor, ME, USA

## Abstract

The GeneWeaver bipartite data model provides an efficient means to evaluate shared molecular components from sets derived across diverse species, disease states and biological processes. In order to adapt this model for examining related molecular components and biological networks, such as pathway or gene network data, we have developed a means to leverage the bipartite data structure to extract and analyze shared edges. Using the Pathway Commons database we demonstrate the ability to rapidly identify shared connected components among a diverse set of pathways. In addition, we illustrate how results from maximal bipartite discovery can be decomposed into hierarchical relationships, allowing shared pathway components to be mapped through various parent-child relationships to help visualization and discovery of emergent kernel driven relationships. Interrogating common relationships among biological networks and conventional GeneWeaver gene lists will increase functional specificity and reliability of the shared biological components. This approach enables self-organization of biological processes through shared biological networks.

## Introduction

The GeneWeaver system (http://www.geneweaver.org) offers a web-based platform for the integrated analysis of experimental data across a diversity of species and data types [1,2]. The current production implementation of GeneWeaver captures sets of genes derived from genome and genetic experimentation and annotations of curated community resources, including literature, community ontologies, and user-defined or domain-specific sets. Each unique representation of a gene set is mapped to a defined notion of biological process, disease state, or behavior construct. The bipartite association of genes to larger bio-behavioral assertions allows GeneWeaver to apply a wide variety of combinatorial approaches to interrogate aggregated sets of genes for common genetic components. For example, the GeneWeaver hierarchical similarity tool [3] leverages the application of an exhaustive maximal biclique analysis to identify the inherent hierarchical relationships of shared components in multiple described processes. Discovery of genes related to numerous independently identified but related processes has proven to be a rapid method to discover the convergence of biological processes [4,5].

While the elucidation of common gene components in numerous and diverse sets of genes has proven to be highly valuable for examining the shared relationships in sets of independent process components, it does not explicitly identify gene-gene relationships shared among the same multiple biological processes. Framing this as the common sub-graph problem can successfully identify a set of common edges within a larger set of edges when the nodes have identifiers, as is the case with GeneWeaver. However, in place of traditional approaches, we demonstrate how bipartite graph exploration, as is implemented in GeneWeaver, can be adopted for the discovery of hierarchical edge relationships among a set of networks. This enables the common sub-graphs to be used as intrinsic classifiers for biological processes, concepts or entities for which the network graphs are derived.

Biological pathways represented as network graphs provide an excellent data set for demonstrating the practical implementation of this approach. Here, we use the Pathway Commons data store as a resource from which multiple pathway networks are aggregated. The entire HumanCyc and the curated PID data sets plus portions of the Reactome data set provide a total of 277 pathways with 116103 discrete genes and 155152 gene-to-gene edges. We show that Maximal bicliques in the representative graph can recapitulate shared graph components in the separate pathway resources. In addition, we show that a single shared relationship among many pathways can be hierarchically explored. The ultimate goal is to integrate relationship data into GeneWeaver's model in order to enable refined functional specificity during user-driven convergent data analysis on user-defined collections of graphs.

## Methods

### Data

Biological network data was retrieved from the Pathway Commons database [6] as bulk downloads of available triple relationships in SIF-RDF format. While the Pathway Commons database is designed as a repository for numerous public pathway databases, we choose to retrieve data from the Reactome [7] (Neuronal System, Signal Transduction, and Metabolism components), curated NCI PID [8] and HumanCyc [9] data sets.

**Figure 1:**
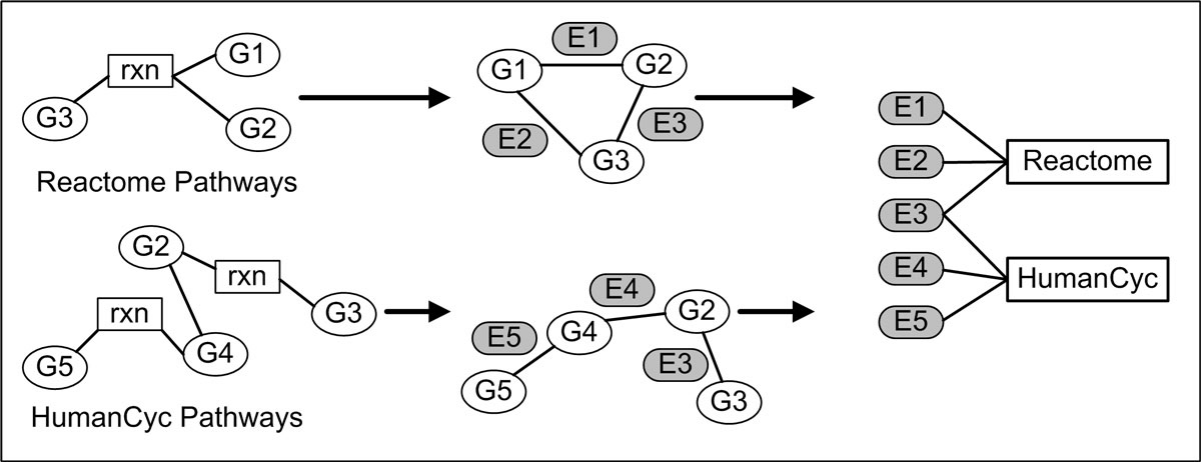
**Pathway network decomposition**. Pathway networks from Pathway Commons were decomposed to discrete gene-to-gene relationships. Edges between related genes are assigned unique ids corresponding to their associated nodes and associated with shared pathways as large bipartite graphs. These graphs were then analyzed to enumerate all maximal bicliques.

GeneWeaver addresses cross-species data harmonization by assigning a unique identifier to clusters associated with biological components, such as genes or transcripts. An extensive, cross species repository of identifier clusters allows each node to be mapped onto a known GeneWeaver identifier, such as the UniProt URI's [10] available from Pathway Commons. Pathways of interest were decomposed from native RDF relationships to lists of associated nodes, representing connected biological components [Figure 1]. The process for transposing RDF to a pairwise graph structure required the application of external reference lists of pathways of interest to seed the graph search. For each pathway, the resulting condensed graphs only contained relationships between genes that were either (a) directly connected or (b) connected through a relationship that could be interpolated as a contiguous uninterrupted path between two genes. A unique edge id is assigned to these GeneWeaver translated node-node associations, creating a list of edges, each associated with a pathway network of interest. Lists of edges operate as components in the bipartite graph analysis toolset, as previously described [3,11].

### Analysis

**Figure 2:**
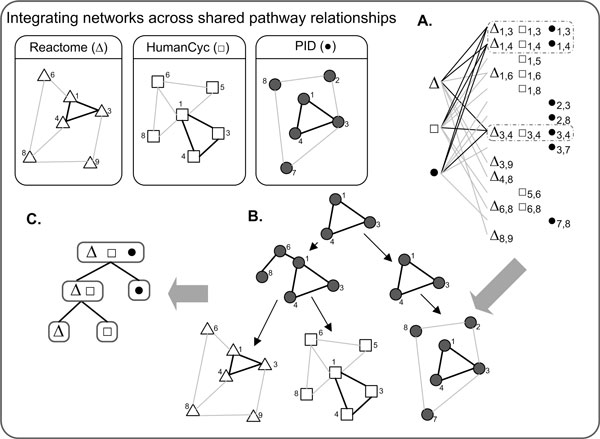
**Hierarchical graph similarity relationships**. Independent pathway networks are converted to large bipartite graphs. The discovery of a maximal bipartite graph represents the highest (top) node of aggregation (A, B). The maximal bipartite decompositions of this node can be described as a hierarchical representation of the (B) bipartite graph components, or shared subgraphs in this case, and the (C) hierarchical relationship of the original pathways.

Rapid bipartite analysis allows for the real time discovery of maximal biclques in very large bipartite graphs. After decomposing pathway networks into discretized bipartite graphs, we can efficiently identify hierarchical relationships of conserved edges among lists of edges [Figure 2]. This allows a user to identify common connected components in a variety of networks, spanning species, network types, and independently derived lists of nodes.

### Visualization

Graphs are built from the edges in each maximal biclique. Because each set of edges and its consequent graph is most likely not completely connected, the largest connected component of each graph is calculated. To find the largest connected component of each graph, a simple breadth first search algorithm is used. This algorithm runs in linear time in terms of the number of vertices and edges since the graph is represented by an adjacency list. The algorithm finds one connected component at a time by doing a breadth first search on each node that has not been found in a previous search, until there are no more unknown nodes.

The graphs are then hierarchically arranged, placing the largest connected component of the maximal biclique at the top level of the graph and all connected components submaximal intersection on lower levels. In order to aid in visualization, each biclique is assigned a color and the nodes in this graph are colored according to the highest hierarchical graph to which they belong. This creates a key for the relevance of specific nodes in a network. Before finding the largest connected component of the largest graph, the edges of graphs within the two levels below the graph are added to make the root graph have interpolated edges.

## Results

**Figure 3:**
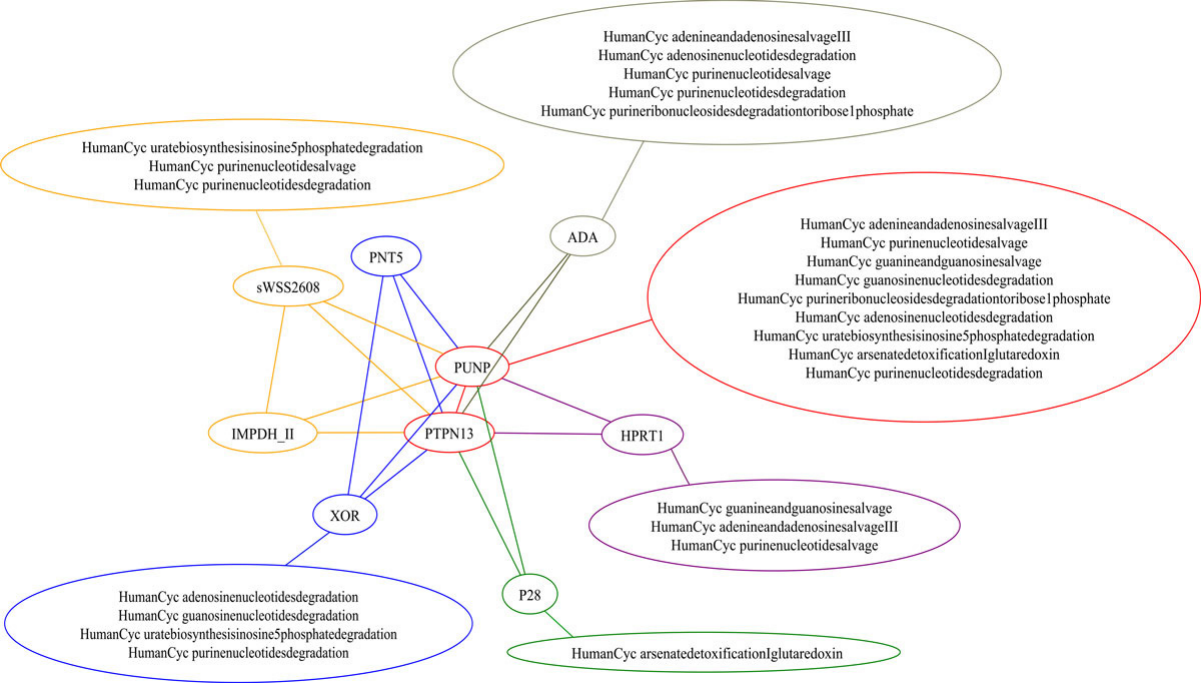
**Discovery of shared sub-graphs in the HumanCyc Pathway dataset**. The common Punp-PtpN13 interaction is seen in nine distinct pathways: adenine and adenosine salvage III, purine nucleotide salvage, guanine and guanosine salvage, guanosine nucleotides degradation, purine ribonucleosides degradation to ribose 1 phosphate, adenosine nucleotides degradation, urate biosynthesis, arsenate detoxification, purine nucleotides degradation. This core pathway is augmented by the addition of edges common to submaximal bicliques, providing a more granular differentiation of the HumanCyc pathway subsets into salvage, degradation pathway, and detoxification-specific pathways, as seen in this example.

An analysis of maximal bicliques is capable of discovering conserved pathways within the same data source, such as the HumanCyc data set. [Figure 3] illustrates one such maximal biclique that represents a common sub-pathway represented in nine HumanCyc pathway graphs. The point of highest convergence is an edge between Punp and PTPN13. Edges unique to submaximal bicliques are colored according to the sets of graphs that they have in common.

**Figure 4:**
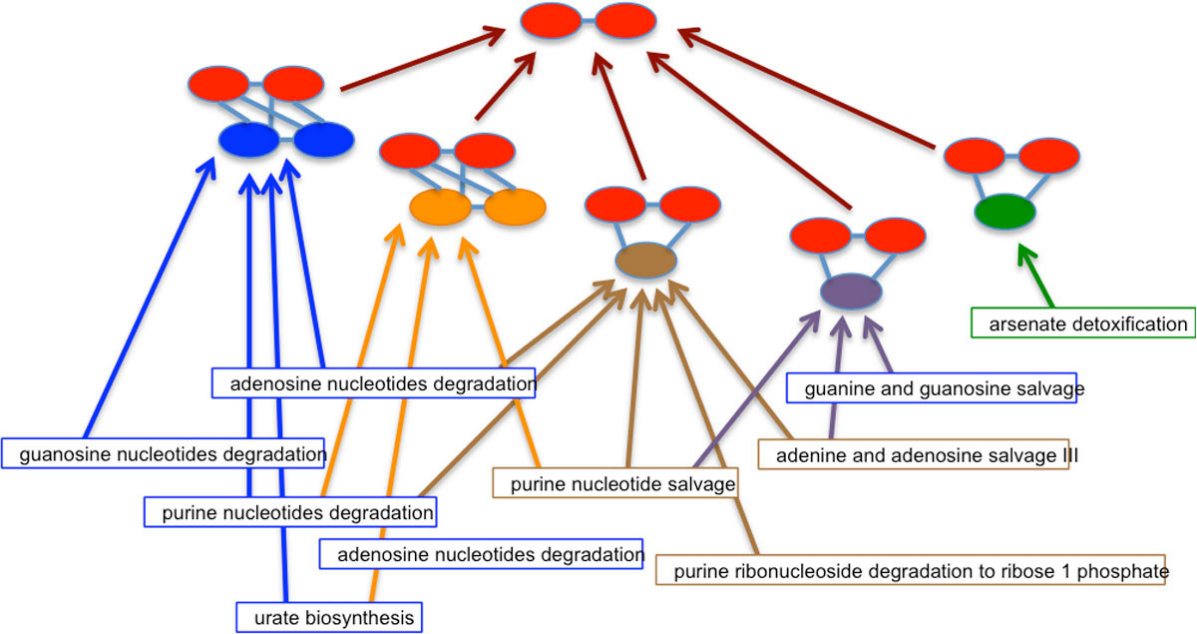
**A hierarchical view of the maximal bipartite interaction graph**. The graph illustrated in Figure 3 is rendered with the common sub-graph at the top node. Each intersecting node below the top node represents a maximal bipartite intersection for the included pathways. This allows common and distinct sub-graphs to be distinguished between different pathways.

[Figure 4] renders the graph shown in [Figure 3] as a hierarchical graph, with the common sub-graph represented at the top node. The hierarchical view of common pathways creates an emergent ontology of the underlying pathways that may be distinct from their semantic organization.

**Figure 5:**
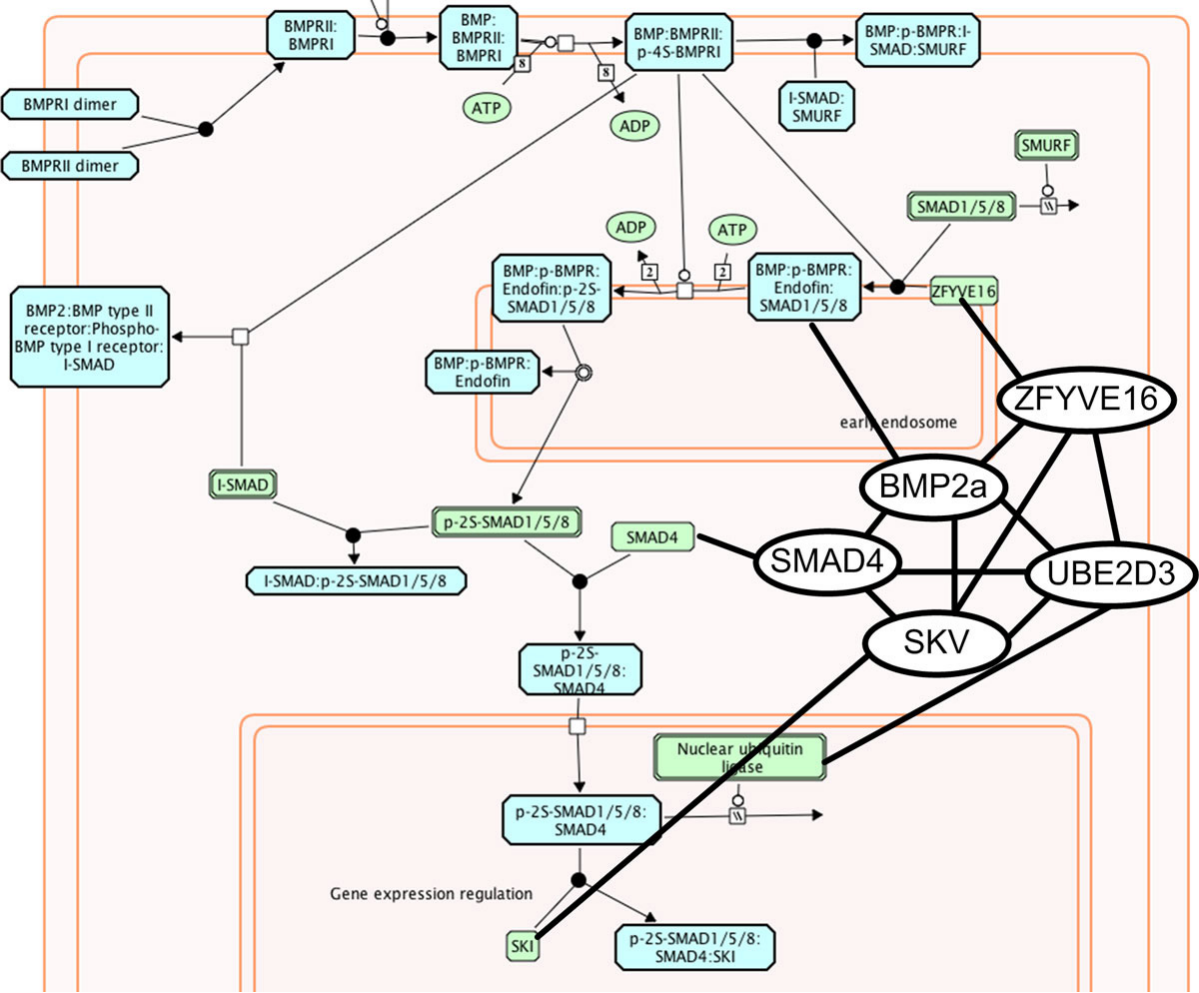
**Shared sub-graphs at the intersection of distinct Pathway Commons resources**. Maximal biclique analysis can rapidly isolate sub-pathways that are common between differing data sources, such as the BMP signaling pathway in this example. The Reactome BMP signaling pathway is shown along with the sub-graph in common with the HumanCyc

**Figure 6:**
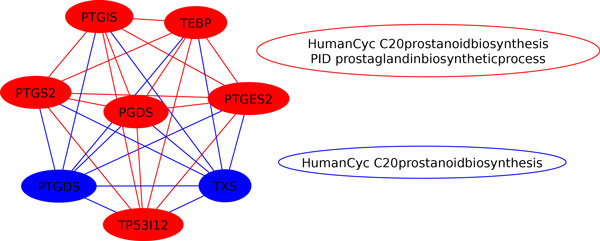
**Shared sub-graphs at the intersection of HumanCyc and PID pathways**. Here, the intersection of the PID prostaglandin biosynthesis and HumanCyc prostanoid biosynthesis pathways are shown in red. Notice that this intersection represents the entire PID prostaglandin biosynthesis pathway. The HumanCyc pathway includes two addition components (blue).

In other cases, the maximal biclique can occur in edges between pathways from different pathway repositories. [Figure 5] demonstrates one such common sub-pathway shared by Reactome and the HumanCyc data set. The sub-pathway is specific to BMP signaling and aligns both pathways based on the maximal biclique shared between them. [Figure 6] illustrates a similar example between the HumanCyc and curated NCI PID data sets. Here, the maximal intersection between the HumanCyc prostanoid biosynthesis pathway and the PID prostaglandin biosynthesis pathway represents the complete PID prostaglandin biosynthesis pathway. The HumanCyc pathway contains two addition gene components.

## Discussion

Finding common sub-graphs in diverse species has the potential to identify pathological states, evolutionary conservation and other common biological processes. While there are several approaches to aligning [12] and mining pathway data [13], they are somewhat restrained by the data structures imposed by RDF. By transposing pathway commons data into a discretized bigraph, our approach makes it accessible to broader set of combinatoric tools, such as maximal biclique analysis. A result of which are hierarchical graph representations of pathway intersections. This provides a means of using shared biology, at the level of network graphs, to identify shared processes and has applications to many problems in biological classification. These include species and cell type identification, disease differentiation, developmental state transitions, ontology alignment, drug discovery and a host of applications in which one might wish to not only define the shared networks among edges, but to sort the graphs (and the biological concepts they represent) based on these shared edges. This unsupervised classification will enable end users of the GeneWeaver system to search a collection of network graphs, and/or provide their own, enumerate the core underlying biology and identify subset concepts among them.

There are many challenges that remain in the enumeration of shared network subgraphs. Methods for missing nodes and missing edges will reduce noise in the hierarchical decomposition and increase aggregation, i. e. the number of subgraph components in each node of the hierarchical similarity graph. This is particularly critical for the alignment of graphs across species, for which homologous gene mappings will be incomplete, particularly as phylogenetic distances among the source data species is increased.

The bipartite graph representation is a convenient means of specifying the problem of shared network edges, and is related to other graph representations (e.g. hypergraphs) and data mining applications including market basket analysis. The maximal biclique algorithm is highly efficient and capable of fast enumeration of all shared network edges among a very large set of graphs. Starting with LCCs increases the efficiency of the pathway comparison analysis, but it does not necessarily ensure the best or most detailed overlap among network graphs. Performance enhancements, particularly on post-processing of sets of network edges will be required to comprehensively enumerate all shared subgraphs among sets of graphs.

Understanding the conditionality of biological associations across networks and pathways provides a powerful means of interpreting broadly constructed interaction, co-expression and co-occurrence networks. The algorithms and approaches that we have devised provide a means for integrating diverse network data to identify convergent and conditional evidence for the interaction of biological entities and the mapping of those common interactions onto shared process or other contextual information. While the most immediate applications are found in functional genomics, extension is readily possible for cellular, anatomical and evolutionary biology, among other more general graph comparison problems.

## Competing interests

The authors declare that they have no competing interests.

## Notes

This article has been published as part of *BMC Proceedings *Volume 8 Supplement 6, 2014: Proceedings of the Great Lakes Bioinformatics Conference 2014. The full contents of the supplement are available online at http://www.biomedcentral.com/bmcproc/supplements/8/S6.
